# Mechanism of Datura metel on sinus bradycardia based on network pharmacology and molecular docking

**DOI:** 10.1097/MD.0000000000032190

**Published:** 2022-12-09

**Authors:** Feifei Yang, Pihong Liu, Xiaosi Zhang, Zhe Zhang, Hao Lu, Naizhi Geng

**Affiliations:** a Heilongjiang University of Chinese Medicine, Harbin, China; b First Affiliated Hospital of Heilongjiang University of Chinese Medicine, Harbin, China; c Beijing University of Chinese Medicine, Bingjing, China; d Affiliated Hospital of North Sichuan Medical College, Nanchong, China; e Tianshui City Hospital of Traditional Chinese Medicine, Tianshui, China

**Keywords:** Datura metel, molecular docking, network pharmacology, sinus bradycardia

## Abstract

To investigate the mechanism of action of Datura metel in the treatment of sinus bradycardia based on network pharmacology and molecular docking.

**Methods:**

The active ingredients and targets of Datura metel were collected by TCMSP database, and the Cytoscape software was used to map to show the interrelationship. Use 5 databases: GeneCards, PharmGKB, OMIM, DisGeNET, and Drugbank to obtain targets related to sinus bradycardia; establish a protein-to-protein interaction network with the help of the STRING platform; GO and Kyoto Encyclopedia of Genes and Genomes analysis of the selected core targets using the Metascape platform; Finally, the AutoDock platform was used for molecular docking and the results were displayed through Pymol.

**Results:**

27 kinds of active ingredients of the drug were screened, including 10 kinds of main ingredients; 198 drug targets and 1059 disease targets. There are 54 targets of action in the treatment of sinus bradycardia, of which 19 targets such as AKT1, IL6, TNF, and VEGFA are the core targets of Datura metel in the treatment of sinus bradycardia. Kyoto Encyclopedia of Genes and Genomes obtained 18 results suggesting that AGE-RAGE, hepatitis C, relaxin, and JAK-STAT may be key signaling pathways. Molecular docking shows that most components of the drug have good docking ability with the core target, indicating that the prediction results have certain reliability.

**Conclusion:**

This study preliminarily explores the potential active ingredients and possible mechanisms of action of Datura metel in the treatment of sinus bradycardia and provides a basis for in-depth investigation of its medicinal material basis and mechanism of action.

## 1. Introduction

Sinus bradycardia (SB or DH) refers to a sinus rhythm with a frequency of fewer than 60 beats/min,^[1]^ which is characterized by varying degrees of palpitations, chest tightness, dizziness, weakness, chills and cold extremities, or even syncope, cardiogenic shock, and sudden death. Asymptomatic sinus bradycardia often requires no treatment, but in modern medicine, western drugs such as atropine have short-lived and unstable effects, as well as the high cost of pacemakers.

**Figure 1.**
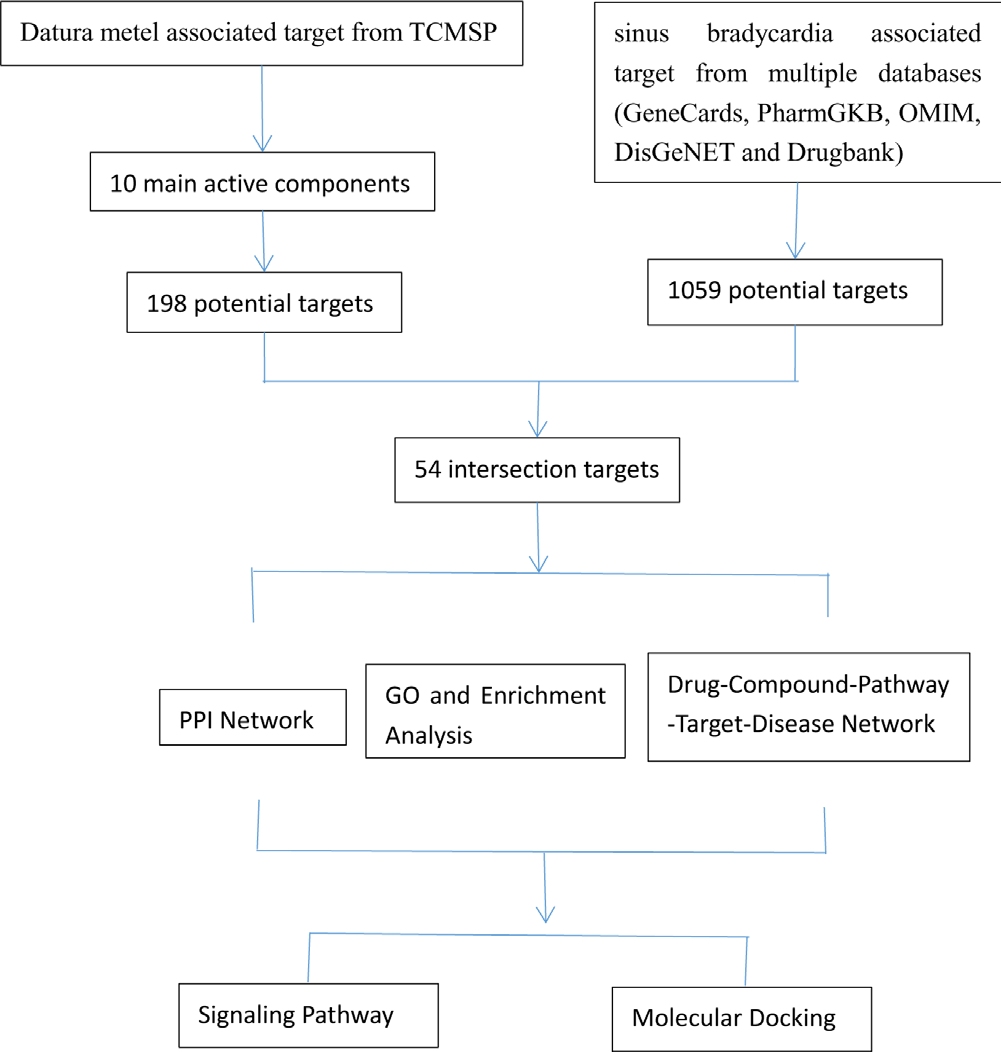
Flowchart of this study.

Chinese medicine is a unique treasure trove of medicine in China and has significant and irreplaceable advantages in the prevention and treatment of many diseases such as sinus bradycardia.^[2]^ Modern pharmacological results have confirmed that scopolamine, the active ingredient of Datura metel (DM or YJH), can increase heart rate better at higher doses,^[3,4]^ and clinical reports of formulas using Datura metel as one of the key drugs to increase sinus rhythm have been increasing year by year. The clinical observation found that the combination of Heart Treasure Pill with Datura metel as the main ingredient and the basic treatment of western medicine can increase the improvement rate of sinus bradycardia with ventricular anterior contraction by more than 15%.^[5]^ In addition, it has been clinically found that the combination of Heart Treasure Pill and Irbesartan can better increase heart rate while improving cardiac function.^[6]^ Although Datura metel is effective in increasing sinus rhythm, few studies use big data systems to deeply explore the mechanisms of its components. From the idea of the holistic concept of traditional Chinese medicine, the author has used modern network pharmacology^[7]^ and molecular docking methods to reveal the main chemical components, targets, and mechanisms of Datura metel to increase heart rate, to provide a more reliable theoretical basis for the use of Datura metel in the treatment of sinus bradycardia. The corresponding workflow is shown in Fig. 1.

## 2. Materials and methods

### 2.1. Data sheet

Web-based pharmacology and molecular docking studies require the use of web-based databases and software platforms, and the URLs of the databases used in this paper are listed in Table 1.

**Table 1:** Database name and URL.

Database name	Database URL
TCMSP	http://tcmspw.com/tcmsp.php
UniProt	http://www.uniprot.org/
GeneCards	https://www.genecards.org/
OMIM	https://omin.org/
DrugBank	https://www.drugbank.ca/
DisGeNET	https://www.disgenet.org/
PharmGkb	https://www.pharmgkb.org/
Venny 2.1	http://bioinfogp.cnb.csic.es/tools/venny/index.html
String	https://string-db.org/
Metascape	http://metascape.org/gp
Bioinformatics	http://swyxzj.com
PDB	https://www.rcsb.org/
PubChem	https://pubchem.ncbi.nlm.nih.gov/

### 2.2. Methods

#### 2.2.1. Screening of active ingredients and target genes of Datura metel.

All the chemical components of Datura metel were firstly obtained from the TCM Systematic Pharmacology (TCMSP) database,^[8]^ and then the active ingredients and related targets of Datura metel were screened with the conditions of oral bioavailability ≥ 30%, and drug-like properties ≥ 0.18. The active ingredients and the corresponding targets were compiled in Excel and entered into the UniProt database^[9]^ one by one, with “Reviewed” and “Human” as constraints, to obtain the gene abbreviations of all targets. After de-duplication, the gene results of the targets of the active ingredients of Datura metel were obtained.

#### 2.2.2. Sinus bradycardia-related target search and intersection target acquisition.

Using “sinus bradycardia” as a keyword, searches were conducted in the 5 classic databases GeneCards,^[10]^ Online Human Mendelian Genetic,^[11]^ DrugBank,^[12]^ DisGeNET,^[13]^ Pharmacogenomics Knowledge Base,^[14]^ and the results were collated in Excel format and deduplicated for the desired The results are then deduplicated to the desired disease target. GeneCards were used to retain results for genes greater than the median. The corresponding targets of the active ingredient and the disease target were imported into Venny2.1^[15]^ to obtain the intersecting targets.

#### 2.2.3. Protein interactions network construction and core target screening for sinus bradycardia treatment with Datura metel.

Protein interactions were analyzed using the String^[16]^ database. The intersecting targets from 2.2.2 were imported into the string database, species were selected as “Homo sapiens” and free nodes were hidden, other parameters were set to default values, PPI networks were constructed, and the “tsv” file was exported. The built-in plug-ins Bisogenet and CytoNCA of Cytoscape 3.7.1^[17]^ were used to filter for degree centrality, proximity centrality, and intermediate centrality to derive core targets.

#### 2.2.4. Gene Ontology (GO) and Kyoto Encyclopedia of Genes and Genomes (KEGG)) signaling pathway enrichment analysis.

GO functional annotation and KEGG pathway enrichment analysis were performed for the intersection targets of Homo sapiens and sinus bradycardia based on the Metascape database.^[18]^ The biological process (BP), cellular component (CC), molecular functions (MF), and KEGG pathways were obtained and collated in Excel format. The results of the data were plotted using the Bioinformatics online mapping website as GO triple bar graphs and KEGG bubble plots.

#### 2.2.5. “Drug-component-pathway-target-disease” network construction.

To visualize the modern pharmacological rationale for the treatment of sinus bradycardia with Datura metel, the Cytoscape 3.7.1 software was used to construct a “drug-key component-pathway-intersection target-disease” network diagram.

#### 2.2.6. Molecular docking validation of “drug component-core target.”

The 3D structures of the top 4 core targets (AKT serine/threonine kinase 1 (AKT1), IL6, vascular endothelial growth factor (VEGFA), tumor necrosis factor (TNF) in the PDB database^[19]^ were obtained in PDB format, and the water molecules and ligands were removed in Pymol 2.4.0 software; the mol2 structures of the top 4 core targets in the PubChem database^[20]^ were obtained in mol2 format; molecular docking was performed by AutoDockTools 1.5.6 software. Docking was performed by Autodock software to obtain the highest binding energy sites; visualization was performed by Pymol software.

## 3. Results and analysis

### 3.1. Results of the collection of constituents and targets of Centaurea

A total of 27 chemical components of Datura metel were obtained in TCMSP (Table 2), and the absorption, distribution, metabolism, excretion (ADME) criteria are shown in Table 3. By searching the 27 components separately, it was found that a total of 10 components had corresponding targets, and the final screening yielded 10 and 198 active components and targets of Datura metel (Table 4).

**Table 2:** The active ingredient of Datura metel.

MOL ID	MOL NAME	OB	DL
MOL011093	Apohyoscine	59.68	0.25
MOL001554	Scopolamine	67.97	0.27
MOL011470	12-Deoxywithastramonolide	109.97	0.75
MOL011484	Datuarmeteloside A	36.13	0.25
MOL011486	Datuarmeteloside B	31.74	0.26
MOL011487	Datuarmeteloside B_qt	62.06	0.75
MOL011488	Datuarmeteloside C	41.16	0.25
MOL011490	Datumetelin	56.04	0.68
MOL011491	Datumetine	84.74	0.18
MOL011495	Daturametelin A_qt	42.04	0.89
MOL011497	(6R)-6-[(1R)-2-hydroxy-1-[(8S,9S,10R,13S,14S,17R)-1-keto-10,13-dimethyl-4,7,8,9,11,12,14,15,16,17-decahydrocyclopenta[a] phenanthren-17-yl]ethyl]-4-methyl-3-methylol-5,6-dihydropyran-2-one	53.86	0.90
MOL011498	(1R,2R,5R,6S)-2-[(8S,9S,10R,13S,14S,17R)-10,13-dimethyl-1-oxo-4,7,8,9,11,12,14,15,16,17-decahydrocyclopenta[a]phenanthren-17-yl]-6 -(methoxymethyl)-5-methyl-4,8-dioxabicyclo[3.3.1]nonan-7-one	81.28	0.68
MOL011499	Daturameteline A	50.40	0.73
MOL011505	Daturameteloside E	36.00	0.37
MOL011507	Daturameteloside F	45.10	0.35
MOL011519	Hyoscine	49.84	0.27
MOL011520	hypaconitine	31.39	0.26
MOL011531	Secowithamerclin	50.21	0.89
MOL011539	Withametelin	83.59	0.77
MOL011540	withanolide D	58.29	0.76
MOL003283	(2R,3R,4S)-4-(4-hydroxy-3-methoxy-phenyl)-7-methoxy-2,3-dimethylol-tetralin-6-ol	66.51	0.39
MOL003644	Withaferine	33.14	0.73
MOL000422	Kaempferol	41.88	0.24
MOL005406	Atropine	45.97	0.19
MOL000631	Coumaroyltyramine	112.90	0.20
MOL007923	2-(4-hydroxyphenyl)ethyl (E)-3-(4-hydroxyphenyl)prop-2-enoate	93.36	0.21
MOL000098	Quercetin	46.43	0.28

**Table 3:** ADME-Tox prediction.

Mol ID	Molecule Name	MW	AlogP	Hdon	Hacc	OB (%)	Caco-2	BBB	DL	FASA-	HL
MOL011093	Apohyoscine	285.37	1.93	0	4	59.68	0.84	0.68	0.25	0.33	2.71
MOL001554	Scopolamine	303.39	0.82	1	5	67.97	0.37	-0.01	0.27	0.3	4.16
MOL011470	12-Deoxywithastramonolide	472.68	3.2	2	6	109.97	-0.02	-0.48	0.75	0.24	6.34
MOL011484	Datuarmeteloside A	650.84	0.36	6	12	36.13	-1.54	-2.09	0.25	0.23	10.7
MOL011486	Datuarmeteloside B	632.82	1.43	5	11	31.74	-1.3	-1.94	0.26	0.26	10.5
MOL011487	Datuarmeteloside B_qt	470.66	3.18	2	6	62.06	0.03	-0.44	0.75	0.26	6.85
MOL011488	Datuarmeteloside C	648.82	0.33	6	12	41.16	-1.71	-2.42	0.25	0.27	10.6
MOL011490	Datumetelin	468.69	3.64	0	5	56.04	0.52	0.11	0.68	0.25	4.88
MOL011491	Datumetine	275.38	2.3	0	4	84.74	0.99	0.85	0.18	0.29	3.16
MOL011495	Daturametelin A_qt	438.66	5.11	1	4	42.04	0.37	-0.12	0.89	0.28	6.67
MOL011497	(6R)-6-[(1R)-2-hydroxy-1-[(8S,9S,10R,13S,14S,17R)-1-keto-10,13-dimethyl-4,7,8,9,11,12,14,15,16,17-decahydrocyclopenta[a]phenanthren-17-yl]ethyl]-4-methyl-3-methylol-5,6-dihydropyran-2-one	454.66	3.89	2	5	53.86	-0.01	-0.73	0.9	0.26	8.14
MOL011498	(1R,2R,5R,6S)-2-[(8S,9S,10R,13S,14S,17R)-10,13-dimethyl-1-oxo-4,7,8,9,11,12,14,15,16,17-decahydrocyclopenta[a]phenanthren-17-yl]-6-(methoxymethyl)-5-methyl-4,8-dioxabicyclo[3.3.1]nonan-7-one	468.69	3.64	0	5	81.26	0.46	0.01	0.68	0.26	5.45
MOL011499	Daturameteline A	486.66	2	3	7	50.4	-0.52	-0.95	0.73	0.3	7.33
MOL011505	Daturameteloside E	650.84	0.67	7	12	36	-1.6	-2.43	0.37	0.27	12.04
MOL011507	Daturameteloside F	666.84	-0.43	8	13	45.1	-2.05	-2.79	0.35	0.27	12.48
MOL011519	Hyoscine	303.39	0.82	1	5	49.84	0.21	-0.19	0.27	0.3	3.44
MOL011520	Hypaconitine	615.79	-0.1	2	11	31.39	-0.15	-0.4	0.26	0.19	22.36
MOL011531	Secowithamerclin	468.69	4.3	1	5	50.21	0.27	-0.2	0.89	0.27	7.07
MOL011539	Withametelin	436.64	4.34	0	4	83.59	0.65	0.37	0.77	0.3	6.38
MOL011540	Withanolide D	454.66	4.34	1	5	58.29	0.16	-0.58	0.76	0.29	6.02
MOL003283	(2R,3R,4S)-4-(4-hydroxy-3-methoxy-phenyl)-7-methoxy-2,3-dimethylol-tetralin-6-ol	360.44	2.25	4	6	66.51	-0.2	-1.17	0.39	0.23	1.26
MOL003644	Withaferine	470.66	3.25	2	6	33.14	-0.21	-1.07	0.73	0.3	6.1
MOL000422	Kaempferol	286.25	1.77	4	6	41.88	0.26	-0.55	0.24	0	14.74
MOL005406	Atropine	289.41	1.72	1	4	45.97	0.43	0.01	0.19	0.28	2.75
MOL000631	Coumaroyltyramine	283.35	2.88	3	4	112.9	0.6	-0.22	0.2	0.41	5.63
MOL007923	2-(4-hydroxyphenyl)ethyl (E)-3-(4-hydroxyphenyl)prop-2-enoate	284.33	3.52	2	4	93.36	0.68	-0.28	0.21	0.42	5.24
MOL000098	Quercetin	302.25	1.50	5	7	46.43	0.05	-0.77	0.28	0.38	14.40

**Table 4:** Components and corresponding targets.

Ingredients	Target name
Apohyoscine	CHRM3, CHRM1, DRD5, SCN5A, CHRM5, ADRA2C, CHRM4, OPRD1, HTR2A, SLC6A2, ADRA1A, HTR2C, CHRM2, ADRA2B, ADRA1B, SLC6A3, ADRB2, SLC6A4, DRD2, OPRM1, GABRA1, DPP4, CHRNA7
Datumetine	drd1, adrb1, scn10a, adra2a
Daturametelin A_qt	PGR, NR3C1
Withametelin	NR3C2
(2R,3R,4S)-4-(4-hydroxy-3-methoxy-phenyl)-7-methoxy-2,3-dimethylol-tetralin-6-ol	ESR1, AR, PPARG, F10, PTGS2, NOS3, CA2, F7, TOP2A, ESR2, MAPK14, GSK3B, HSP90AB1, CHEK1, PIM1, NCOA2, CAMKK2, CCNA2, PTGS1, IGHG1
Kaempferol	NOS2, PIK3CG, PRKACA, PRSS1, F2, GABRA2, ACHE, TOP2A, RELA, IKBKB, AKT1, BCL2, BAX, TNF, JUN, AHSA1, CASP3, MAPK8, XDH, MMP1, STAT1, CDK1, HMOX1, CYP3A4, CYP1A2, CYP1A1, ICAM1, SELE, VCAM1, NR1I2, CYP1B1, ALOX5, HAS2, GSTP1, AHR, PSMD3, SLC2A4, NR1I3, INSR, DIO1, PPP3CA, GSTM1, GSTM2, AKR1C3, SLPI
Atropine	HTR1B, HRH1, HTR1A
Coumaroyltyramine	LTA4H, MAOB, PKIA
2-(4-hydroxyphenyl)ethyl (E)-3-(4-hydroxyphenyl)prop-2-enoate	PPARG
Quercetin	AKR1B10, KCNH2, MMP3, RXRA, EGFR, VEGFA, CCND1, BCL2L1, FOS, CDKN1A, EIF6, CASP9, PLAU, MMP2, MMP9, MAPK1, IL10, EGF, RB1, IL6, CHEK1, TP53, ELK1, NFKBIA POR, ODC1, CASP8, TOP1, RAF1, SOD1, PRKCA, HIF1A, RUNX1T1, HSPA5, ERBB2, ACACA, CAV1, MYC, F3, GJA1, IL1B, CCL2, PTGER3, CXCL8, PRKCB, BIRC5, DUOX2 HSPB1, TGFB1, SULT1E1, MGAM, IL2, CCNB1, PLAT, THBD, SERPINE1, COL1A1, IFNG, PTEN, IL1A, MPO, NCF1, ABCG1, NFE2L2, NQO1, PARP1, COL3A1, CXCL11, CXCL2, DCAF5, CHEK2, CLDN4, PPARA, PPARD, HSF1, CRP, CXCL10, CHUK, SPP1, RUNX2, RASSF1, E2F1, E2F2, ACP3, CTSD, IGFBP3, IGF2, CD40LG, IRF1, ERBB3, PON1, PCOLCE npepps, hk2, nkx3-1, rasa1

### 3.2. Sinus bradycardia target collection and intersection target acquisition results

**Figure 2.**
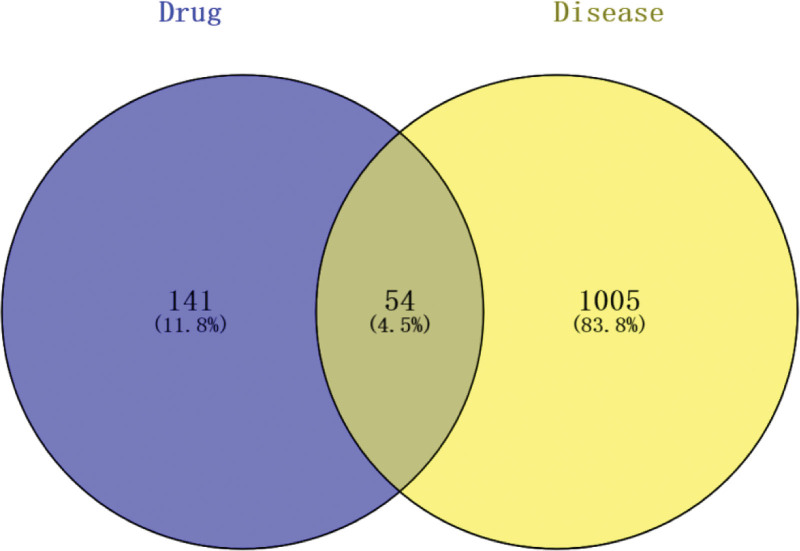
The intersection of Datura metel sinus bradycardia targets.

Retrieved from 5 major databases, GeneCards, Online Human Mendelian Genetic, DrugBank, DisGeNET, and Pharmacogenomics Knowledge Base, respectively, “sinus bradycardia “277, 20, 9, 900, and 114 corresponding targets were obtained, and 1059 targets remained after all targets were combined and deweighted. The intersecting targets were obtained by importing the drug targets and disease targets together into Venny2.1, suggesting that the 54 intersecting targets (Fig. 2) may be the main targets for the treatment of sinus bradycardia with Datura metel.

### 3.3. Protein interaction network map and core target screening results

**Figure 3.**
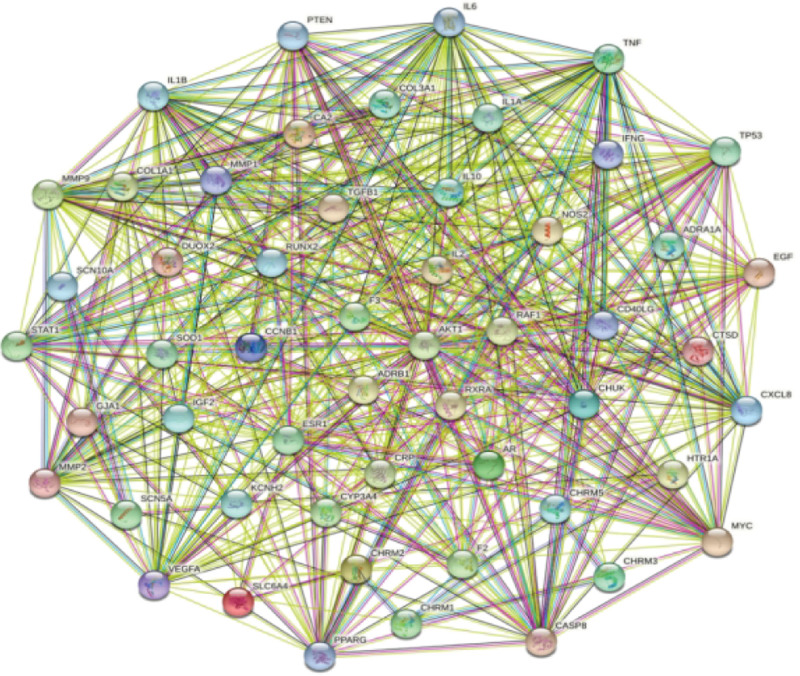
Intersectional target interaction network. The more a target is connected to other targets, the more important the target is.

**Figure 4.**
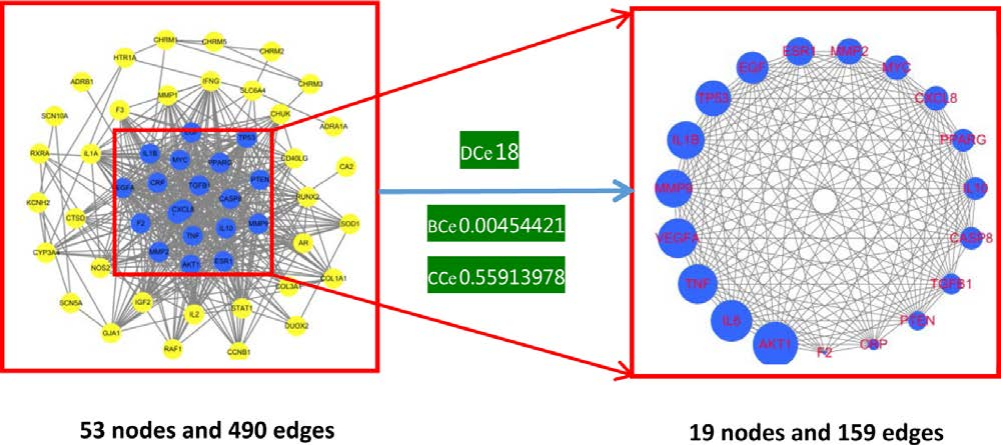
Core target screening. Note: The box on the right is the core target, where the larger the node, the higher the degree value.

The 54 intersecting targets were imported into the String bioanalysis platform, and the PPI network after hiding 1 free node (Fig. 3), which showed a total of 54 nodes and 490 edges (the expected number of edges was 182), with an average degree value of 18.1 per node; then speaking of importing Cytoscape 3.7.1 software, with the help of Bisogenet and CytoNCA for degree centrality, near-centrality, and mediated centrality were screened, resulting in 19 nodes and 159 edges (Fig. 4), suggesting that these 19 nodes are the core targets of Datura metelbe for sinus bradycardia (Table 5).

**Table 5:** Information about the core target of the treatment of sinus bradycardia.

Serial number	Core targets	Protein name	Degree
1	AKT1	RAC-alpha serine/threonine-protein kinase	40
2	IL6	Interleukin-6	38
3	VEGFA	Vascular endothelial growth factor A	37
4	TNF	Tumor necrosis factor	37
5	IL1B	Interleukin-1 beta	36
6	MMP9	Matrix metalloproteinase-9	36
7	TP53	Cellular tumor antigen p53	35
8	EGF	Pro-epidermal growth factor	33
9	ESR1	Estrogen receptor	31
10	MMP2	72 kDa type IV collagenase	30
11	MYC	Myc proto-oncogene protein	29
12	CXCL8	Interleukin-8	29
13	IL10	Interleukin-10	28
14	CASP8	Caspase-8	28
15	PPARG	Peroxisome proliferator activated receptor gamma	28
16	TGFB1	Transforming growth factor beta-1	27
17	PTEN	Phosphatidylinositol-3,4,5-trisphosphate 3-phosphatase and dual-specificity protein phosphatase PTEN	26
18	CRP	C-reactive protein	22
19	F2	Thrombin	18

### 3.4. GO and KEGG signaling pathway enrichment results

#### 3.4.1. GO enrichment analysis.

**Figure 5.**
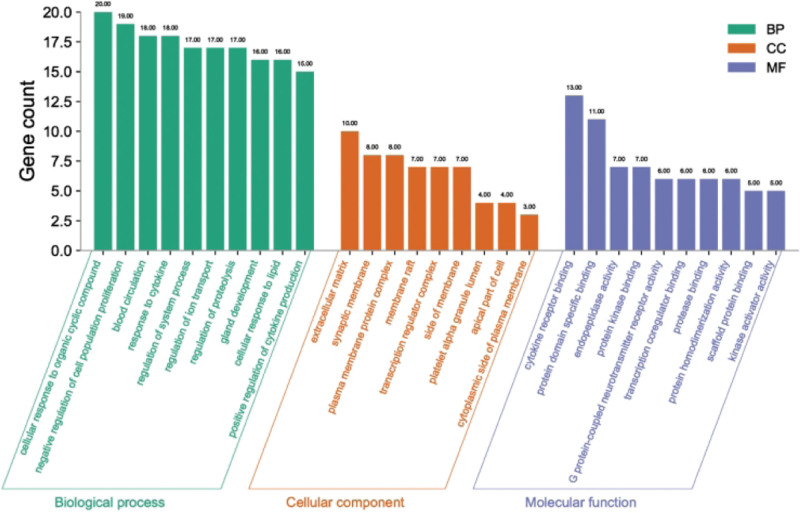
GO analysis results. Note: The x-axis demonstrates the top 9-10 significantly enriched BP, CC, and MF categories, while the y-axis shows the number of enriched genes for these terms (*P* < .05). GO = gene ontology, BP = biological process, CC = cellular component, MF = molecular function.

A total of 4443 GO entries (*P* < .01) were identified by importing 54 intersecting targets into the Metascape database, with the species selected as “Homo sapiens.” Of these, 3700 entries were for BP, mainly related to the cellular response to organic cyclic compound, negative regulation of cell population proliferation, blood circulation, response to cytokine, and regulation of system process. There are 285 entries in CC, mainly dealing with the extracellular matrix, synaptic membranes, plasma membrane protein complex, membrane raft, transcription regulator complex, and other CCs. MF has 458 entries, mainly related to cytokine receptor binding, protein domain specific binding, endopeptidase activity, protein kinase binding, G protein-coupled neurotransmitter receptor activity, and other MFs. The top 10 of BP, the top 9 of CC, and the top 10 of MF were taken for display (Table 6 and Fig. 5).

**Table 6:** GO enrichment analysis.

GOterm	Subgroup	Gene count
Cellular response to organic cyclic compound	Biological processes	20
Negative regulation of cell population proliferation	Biological processes	19
Blood circulation	Biological processes	18
Response to cytokine	Biological processes	18
Regulation of system process	Biological processes	17
Regulation of ion transport	Biological processes	17
Regulation of proteolysis	Biological processes	17
Gland development	Biological processes	16
Cellular response to lipid	Biological processes	16
Positive regulation of cytokine production	Biological processes	15
Extracellular matrix	Cellular components	10
Synaptic membrane	Cellular components	8
Plasma membrane protein complex	Cellular components	8
Membrane raft	Cellular components	7
Transcription regulator complex	Cellular components	7
Side of membrane	Cellular components	7
Platelet alpha granule lumen	Cellular components	4
Apical part of cell	Cellular components	4
Cytoplasmic side of plasma membrane	Cellular components	3
Cytokine receptor binding	Molecular functions	13
Protein domain specific binding	Molecular functions	11
Endopeptidase activity	Molecular functions	7
Protein kinase binding	Molecular functions	7
G protein-coupled neurotransmitter receptor activity	Molecular functions	6
Transcription coregulator binding	Molecular functions	6
Protease binding	Molecular functions	6
Protein homodimerization activity	Molecular functions	6
Scaffold protein binding	Molecular functions	5
Kinase activator activity	Molecular functions	5

#### 3.4.2. KEGG pathway analysis.

**Figure 6.**
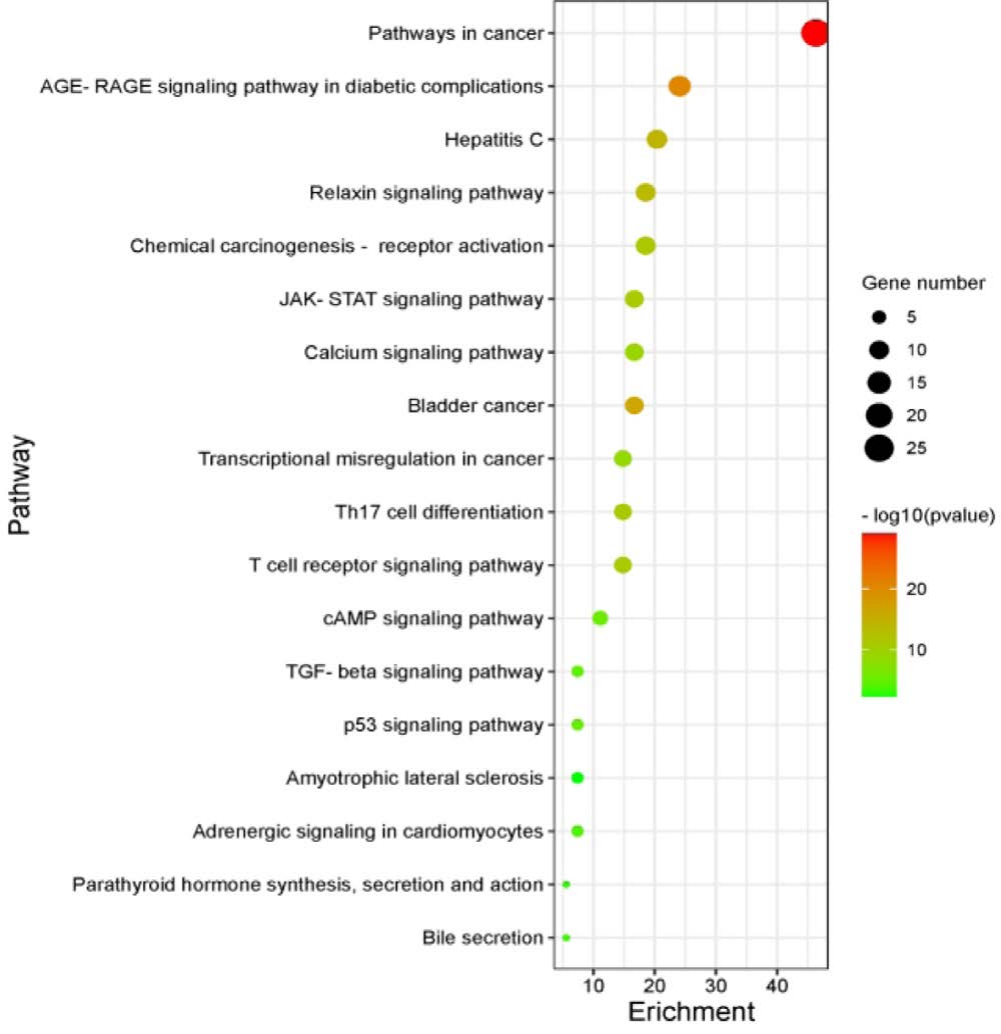
Bubble map of 18 KEGG pathway. Note: The y-axis demonstrates the top 18 significantly enriched KEGG pathways, while the x-axis shows the number of enriched genes for these terms (*P* < .05), the colors and the sizes indicate different *P* value ranges; the redder and bigger it is, the more significantly enriched it is. KEGG = Kyoto Encyclopedia of Genes and Genomes.

**Figure 7.**
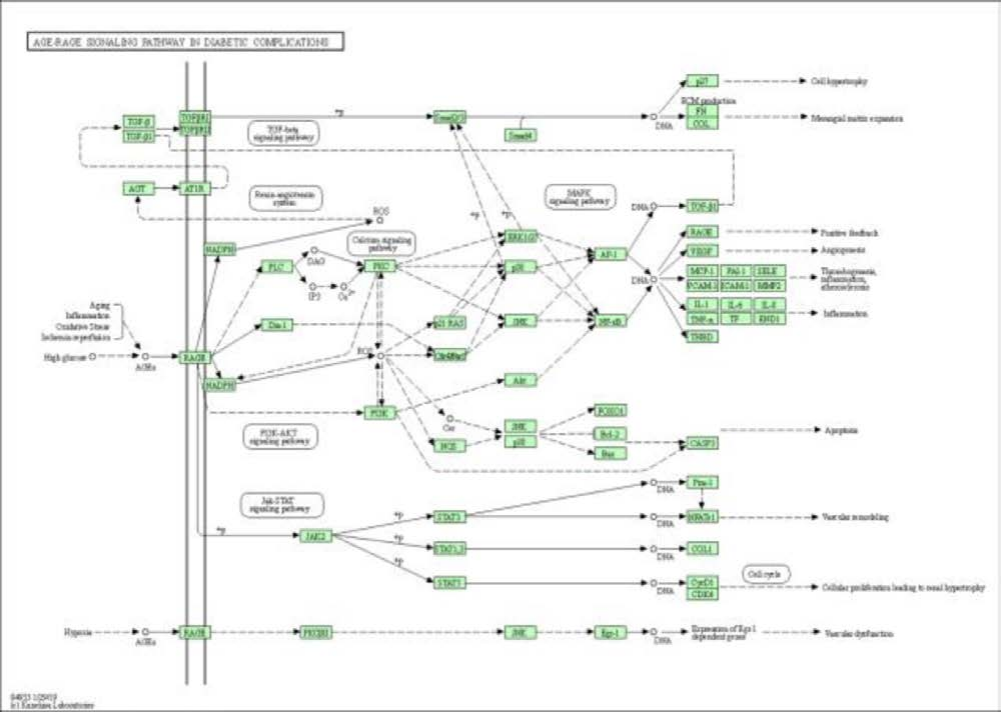
Signal pathway targets of Datura metel in the treatment of sinus bradycardia.

The 54 intersecting targets were imported into the Metascape database, and the species selected was “Homo sapiens,” and KEGG enrichment analysis was performed. 209 key signaling pathways were identified (*P* < .01), of which the first 18 were significant pathways as shown in Table 7 and Fig. 6, mainly involving Pathways in cancer, AGE-RAGE signaling pathway in diabetic complications, hepatitis C, relaxin signaling pathway, and JAK-STAT signaling pathway, etc. Among them, the Calcium signaling pathway, a possible important pathway of Datura metel for sinus bradycardia, is shown in detail in Fig. 7, where the green area in the pathway diagram is signal down-regulation.

**Table 7:** KEGG signalling pathway.

Serial number	Passage ID	Description	Corrected *P*-value	Number of genes
1	Hsa05200	Pathways in cancer	7.94328E-30	25
2	Hsa04933	AGE-RAGE signaling pathway in diabetic complications	2.51189E-21	13
3	Hsa05219	Bladder cancer	3.0903E-17	9
4	Hsa05160	Hepatitis C	4.16869E-15	11
5	Hsa04926	Relaxin signaling pathway	2.88403E-14	10
6	Hsa05207	Chemical carcinogenesis—receptor activation	4.2658E-12	10
7	Hsa04630	JAK-STAT signaling pathway	1.25893E-11	9
8	Hsa04660	T cell receptor signaling pathway	1.38038E-11	8
9	Hsa04659	Th17 cell differentiation	1.86209E-11	8
10	Hsa04020	Calcium signaling pathway	4.2658E-10	9
11	Hsa05202	Transcriptional misregulation in cancer	1.86209E-09	8
12	Hsa04024	cAMP signaling pathway	2.75423E-06	6
13	Hsa04115	p53 signaling pathway	9.12011E-06	4
14	Hsa04350	TGF-beta signaling pathway	2.45471E-05	4
15	Hsa04261	Adrenergic signaling in cardiomyocytes	0.000151356	4
16	Hsa04976	Bile secretion	0.000549541	3
17	Hsa04928	Parathyroid hormone synthesis, secretion and action	0.000912011	3
18	Hsa05014	Amyotrophic lateral sclerosis	0.004073803	4

### 3.5. “Drug-component-pathway-target-disease” network diagram

**Figure 8.**
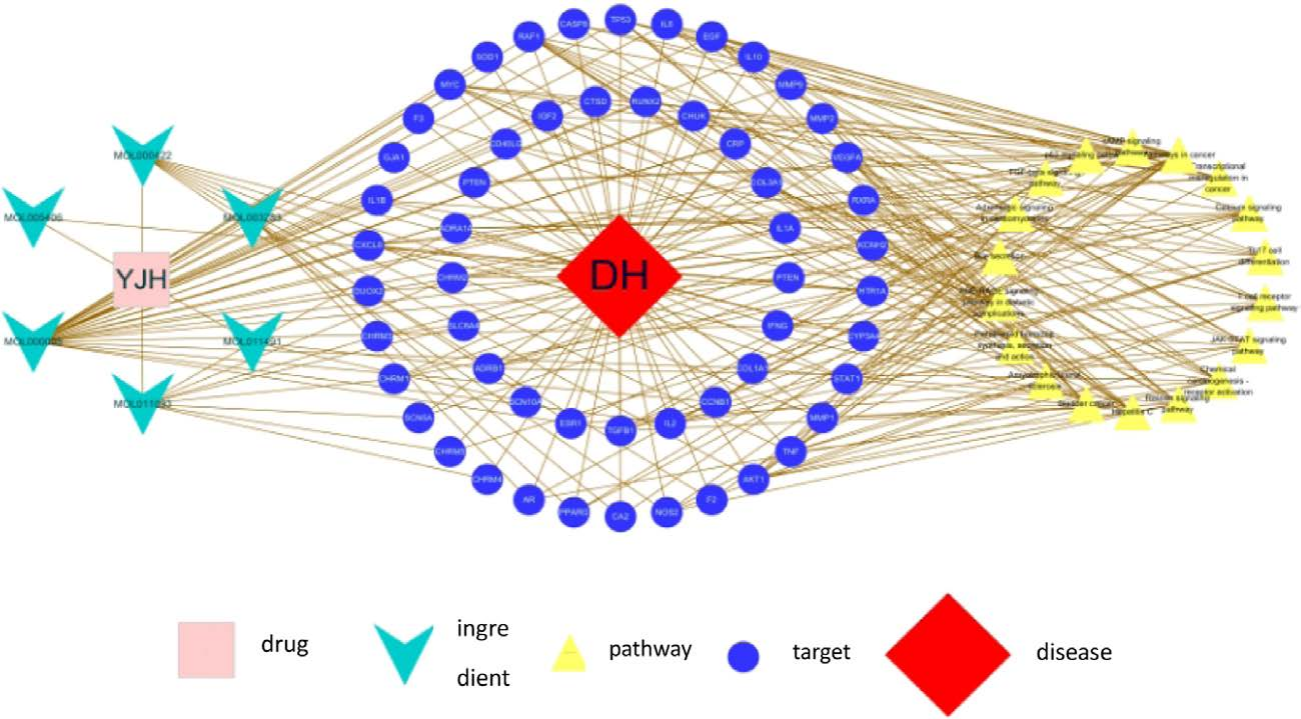
“Drug-component-pathway-target-disease” network diagram. Note: Different colors represent different information. Chocolate yellow represents the medicinal Datura metel, light green color indicates the active active ingredient of the drug, red indicates the disease sinus bradycardia, dark blue represents disease targets, and yellow represents the pathway of action of the drug in the treatment of the disease.

The interrelationship data files and attribute files of drug and key components, key components and intersecting targets, intersecting targets and pathways, and intersecting targets and diseases were prepared in Excel, and both were imported into Cytoscape 3.7.1 line optimization to obtain the final visual network (Fig. 8), where the more lines a node has with other nodes indicates the more important the node is in the network.

### 3.6. Molecular docking results of the active ingredients of Datura metel for sinus bradycardia

**Figure 9.**
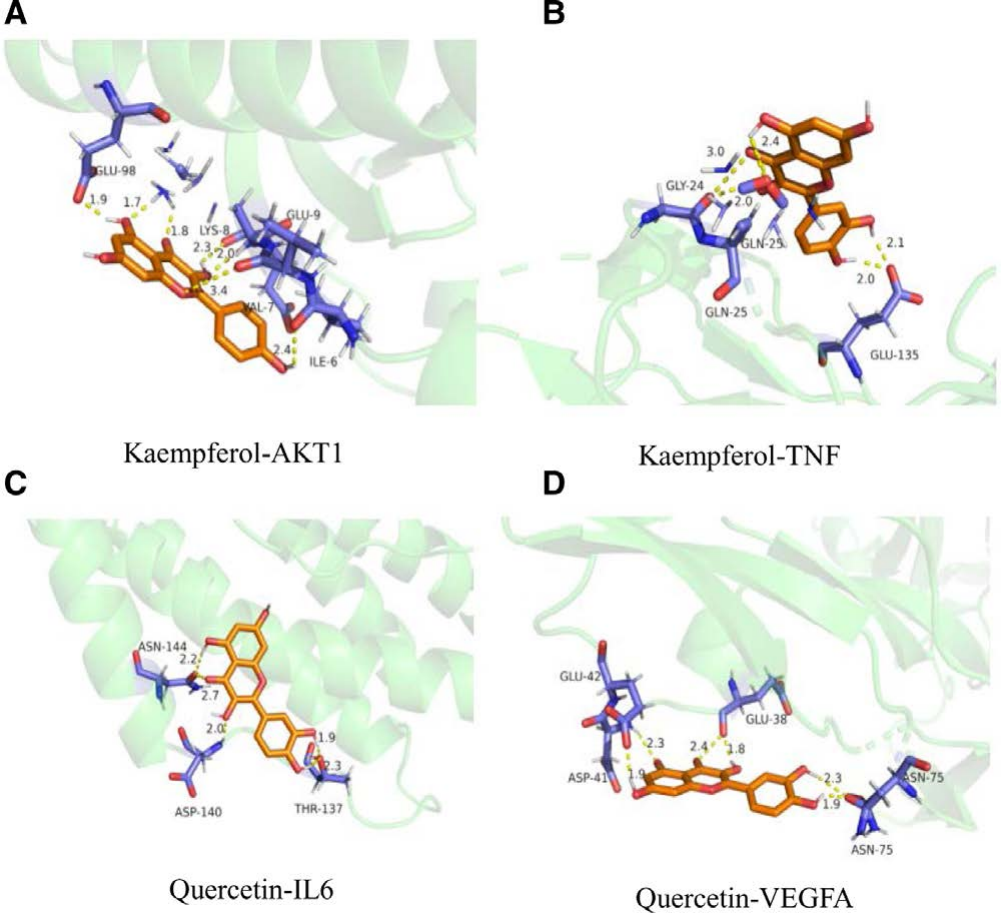
Molecular docking of major component targets.

Since kaempferol and quercetin are the 2 most important components of Datura metel, and AKT1, TNF, IL6, and VEGFA are the top 4 most important ranked core targets screened by degree value among the intersection targets of drug disease, so the molecular docking validation of the most important components and the most important targets has better credibility. Molecular docking of the top 4 Degree values of the core targets and their corresponding components was carried out by AutoDock software and the binding energy is shown in Table 8. The force field used for the energy minimization of small molecule ligands in this study mainly used the MMFF94 force field. In molecular docking, the results were imported into pymol software for visualization and the molecular docking results showed that hydrogen bonding is the main force in the interaction. Kaempferol forms hydrogen bonds with AKT1 at positions GLU-98, GLU-9, LYS-8, VAL-7, and ILE-6; Kaempferol forms hydrogen bonds with TNF at positions GLY-24, GLN-25, GLU-135; quercetin forms hydrogen bonds with IL6 at positions ASN-144, ASP-140, THR-137 positions; quercetin forms hydrogen bonds with VEGFA at GLU-42, GLU-38, ASP-41, ASN-75, and see Fig. 9 for details.

**Table 8:** The binding energy of main active ingredients and key target proteins.

No. Number	Active ingredients Active ingredient	Protein name Protein name	Combined with the ability to Binding energy (kJ/mol)
Mol000422	Kaempferol	AKT1	−5.45
Mol000422	Kaempferol	TNF	−3.83
Mol000098	Quercetin	IL6	−4.32
Mol000098	Quercetin	VEGFA	−4.8

## 4. Discussion

Sinus bradycardia belongs to the category of “palpitation,” or “chest stuffiness and pains” in Chinese medicine, The causes, mechanisms, and treatment of this disease have been described in ancient works such as Yellow Emperor’s Classic of Internal Medicine, Treatise on Febrile Diseases. Modern medicine has confirmed that the causes of this disease are^[21]^ mostly increased excitability of the vagus nerve, impaired function of the sinus node itself, numerous organic heart diseases, etc. It is also related to the long-term use of certain drugs. The pathogenesis^[22]^ is usually related to abnormalities in myocardial ion channel function and autonomic dysfunction. The development of sinus bradycardia is complex, with multiple cells and pathways of action interacting with each other, thus forming a complex network system of interactions. Single-target, single-pathway therapy is not effective, while Chinese medicine has the natural qualities of multiple components, multiple targets, and multiple pathways of action, so it can regulate the body systematically. It has been used for a long time to treat illnesses, with the famous doctor Hua Tuo using it as the main ingredient to make mafei san in the late Eastern Han Dynasty. Abroad, it has a wider range of uses,^[23–25]^ including heart disease, convulsions, diarrhea, hemorrhoids, dysmenorrhea, eczema, and toothache. Although the Chinese Pharmacopoeia 2020 edition^[26]^ does not state that the drug can increase heart rate, several studies^[27]^ have shown that important chemical components of the flower, such as quercetin, have a significant effect on improving heart function, and call for quercetin to be taken seriously in the treatment of many cardiovascular diseases. As research into the chemical composition of the drug has become more advanced, clinical formulations using it as a major component to increase sinus heart rate have been frequently reported, with the Heart Treasure Pill^[28–31]^ being widely used clinically. Although there are many reports, the specific molecular regulatory mechanism of the effect of Datura metel on sinus bradycardia remains unclear. In this study, by searching the TCMSP database, we found that quercetin, apohyoscine, kaempferol, datumetine, atropine, and other active ingredients have more target genes and are the main active ingredients of Datura metel. We obtained the interrelationship network between the above components and sinus bradycardia-related targets through network pharmacology and validated quercetin and kaempferol among them by molecular docking.

After screening by oral bioavailability and drug-like properties parameters, 27 active ingredients were obtained, and a total of 10 active ingredients were identified after eliminating those that could not be found as targets. From the “drug-component-pathway-key target-disease network diagram,” it can be seen that there are 6 major active ingredients directly connected to the key targets, accounting for 60% of the total number of corresponding target ingredients, which are flavonoids and alkaloids. Flavonoids are mostly found in the flowers and leaves of the woody herb and are effective in a variety of diseases of the cardiovascular system. In addition to effectively improving the number of episodes of arrhythmias due to myocardial ischemia, they also have multiple effects such as antiatherosclerosis, anticoagulation, antilipid, and vasodilatation,^[32]^ Chen Qiuhong and others suggest that they may protect against ischemia-reperfusion injury by increasing Na+/K+-ATPase activity and thus improve arrhythmias. The compounds have been shown to improve cardiac arrhythmias by increasing Na+/K+-ATPase activity.^[33]^ These compounds have been shown to exert anti-arrhythmic effects on several ion channels, including potassium, sodium, and calcium, in the myocardial cell membrane.^[34]^ Animal studies have reported that flavonoids have a strong effect on the reduction of inward rectifier potassium currents caused by aconitine.^[35]^ Pei et al^[36]^ found that quercetin had a protective effect on myocardial damage caused by adriamycin in mice. This shows that quercetin and kaempferol, as flavonoids in Chinese medicine, play a “multi-component, multi-target” role in the treatment of sinus bradycardia. In addition, alkaloids also have a role in the improvement of sinus bradycardia. In particular, it is widely known that higher doses of atropine can increase heart rate by relieving vagal inhibition.^[37]^ Studies have shown that scopolamine and scopolamine, which are contained in Hypericum, can improve blood microcirculation by blocking α1 and M receptors, increasing cell membrane fluidity, reducing blood viscosity, promoting tissue healing, increasing heart rate, enhancing myocardial contractility, and inhibiting ectopic excitability of the heart.^[38–40]^

The results of the network screening of core targets showed that AKT1, IL6, VEGFA, and TNF were the top 4 core target proteins in terms of degree value, and maybe the most core targets for the treatment of sinus bradycardia with Yohimbe. A study showed that resveratrol targeting AKT1 inhibited inflammatory vesicle activation in cardiomyocytes under sympathetic stress, inhibited β-adrenergic receptor-mediated NLRP3 inflammatory vesicle activation in cardiomyocytes and mouse hearts, and the resulting cardiac inflammation.^[41]^ IL-6 and VEGFA are closely related to the progression of cardiac hypertrophy and coronary artery disease, while VEGFA is widely distributed in the brain, VEGFA is widely distributed in the brain, heart, and other tissue cells, and has an important regulatory role in angiogenesis and atherosclerosis.^[42]^ Studies have shown that MSCs can reduce inflammation in the heart and lungs through TNF signaling.^[43]^ Mathematical modeling of TNF-α overexpression in transgenic mouse hearts has revealed an effect on cardiac arrhythmias.^[44]^ All of the above suggests a strong association between these targets and the development of arrhythmias.

The analysis of the BPs of the key core targets of *C. elegans* revealed that C. elegans was highly correlated with the BPs of blood circulation, regulation of smooth muscle cell proliferation, and response to peptides in the cardiovascular system, which were also reflected in the key targets of *C. elegans* for the treatment of cardiac arrhythmias. The KEGG pathway enrichment results showed that Hepatitis C, AGE-RAGE signaling pathway, relaxin signaling pathway, JAK-STAT signaling pathway, T cell receptor signaling pathway, calcium signaling pathway, p53 signaling pathway, and adrenergic signaling in cardiomyocytes as the main regulatory pathways. This suggests that the same regulatory mechanisms may exist between different diseases. Evidence suggests a relationship between viral invasion and the development of arrhythmias, such as hepatitis B and C.^[45,46]^ Numerous previous studies have shown that relaxin can hinder fibrosis through many cell targets and signaling pathways.^[47]^ The AGE-RAGE signaling pathway was found to affect the migration and differentiation of cardiac fibroblasts, which in turn affected ventricular reconstruction and cardiac function.^[48]^ In addition, the JAK-STAT signaling pathway plays an important role in myocardial ischemia, infarction, myocarditis, and other cardiac injuries, and its blockade is effective in reducing the inflammatory response in the myocardium and preventing the formation of myocardial fibrosis.^[49]^ Some components, such as arginine pressors, can regulate current by affecting the T-type calcium signaling pathway, increasing intracellular calcium ion concentrations, and triggering prolonged action potential duration and differences in the spatial distribution of action potentials, leading to arrhythmias.^[50]^

In summary, through the use of network pharmacology combined with molecular docking, and the comprehensive discussion and analysis of various networks and databases, the composition targets and mechanisms of action of Datura metel. in the treatment of sinus bradycardia through “multi-component, multi-target, and multi-pathway” have been more fully explored. The results of this study should be investigated in cellular or animal studies to provide further ideas and references for basic and clinical research.

## Author contributions

**Conceptualization:** Feifei Yang.

**Data curation:** Pihong Liu, Xiaosi Zhang.

**Formal analysis:** Feifei Yang, Pihong Liu, Xiaosi Zhang.

**Investigation:** Zhe Zhang.

**Methodology:** Feifei Yang, Hao Lu.

**Project administration:** Naizhi Geng.

**Resources:** Naizhi Geng.

**Software:** Feifei Yang.

**Supervision:** Xiaosi Zhang, Zhe Zhang, Hao Lu.

**Validation:** Naizhi Geng.

**Visualization:** Feifei Yang, Xiaosi Zhang, Zhe Zhang, Hao Lu.

**Writing—original draft:** Feifei Yang.

**Writing—review and editing:** Naizhi Geng, Xiaosi Zhang.

## Correction

The article type has been changed from Narrative Review to Observational Study. “A Review” has been removed as the subtitle of the article title.
